# Mechanisms of escape from the PGT128 family of anti-HIV broadly neutralizing antibodies

**DOI:** 10.1186/s12977-016-0241-5

**Published:** 2016-02-02

**Authors:** Stefanie A. Krumm, Hajer Mohammed, Khoa M. Le, Max Crispin, Terri Wrin, Pascal Poignard, Dennis R. Burton, Katie J. Doores

**Affiliations:** Department of Infectious Diseases, King’s College London School of Medicine, Guy’s Hospital, Great Maze Pond, London, SE1 9RT UK; Department of Immunology and Microbial Science, The Scripps Research Institute, La Jolla, CA USA; IAVI Neutralizing Antibody Center, The Scripps Research Institute, La Jolla, CA USA; Center for HIV/AIDS Vaccine Immunology and Immunogen Discovery, The Scripps Research Institute, La Jolla, CA USA; Oxford Glycobiology Institute, Department of Biochemistry, University of Oxford, South Parks Road, Oxford, OX1 3QU UK; Monogram Biosciences, Laboratory Corporation of America(R) Holdings, South San Francisco, CA USA; Ragon Institute of MGH, MIT and Harvard, Cambridge, USA

**Keywords:** HIV-1, Neutralizing antibody, Viral escape, Envelope glycoprotein, N-linked glycosylation

## Abstract

**Background:**

Broadly neutralizing antibodies (bnAbs) directed against the mannose-patch on the HIV envelope glycoprotein gp120 have several features that make them desirable targets for vaccine design. The PGT125-131 bnAb family is of particular interest due to its superior breadth and potency. The overlapping epitopes recognized by this family are intricate and neutralization requires interaction with at least two N-linked glycans (N332/N334, N295 or N301) in addition to backbone-mediated contact with the ^323^IGDIR^327^ motif of the V3 loop. We have recently shown that this bnAb family consists of two distinct antibody classes that can bind alternate arrangements of glycans in the mannose-patch in the absence of N332 thereby limiting viral escape. This led us to further investigate viral resistance and escape mechanisms to the PGT125-131 bnAb family.

**Results:**

Using an escape virus isolated from the PGT125-131 donor as a guide, we show that mutating both the V3 core protein epitope and repositioning critical N-linked glycosylation sites are required to restore neutralization sensitivity. Interestingly, neutralization sensitivity could be restored via different routes for the two distinct bnAb classes within the PGT125-131 family, which may have been important in generating the divergence in recognition. We demonstrate that the observed V3 mutations confer neutralization resistance in other virus strains through both gain-of-function and escape studies. Furthermore, we show that the V3 loop is important in facilitating promiscuous binding to glycans within the mannose-patch.

**Conclusions:**

These data highlight the importance of the V3 loop in the design of immunogens aimed at inducing broad and potent bnAbs that can bind promiscuously to the mannose-patch.

**Electronic supplementary material:**

The online version of this article (doi:10.1186/s12977-016-0241-5) contains supplementary material, which is available to authorized users.

## Background

Design of an immunogen that elicits broadly neutralizing antibodies (bnAbs) against HIV will likely be a key step in the development of an efficacious HIV vaccine [1]. Approximately 10–30 % of HIV infected individuals develop a bnAb response [2, 3] and it has been shown that these bnAbs, when passively administered to macaques, confer protection against SHIV challenge [4–8]. A number of bnAb sites of vulnerability have been identified on HIV Envelope (Env) including the CD4 binding site, N-glycan-associated epitopes on the V1/V2 loops (centred around N160) and the V3 loop (centred around N332) and regions on gp41 including the membrane proximal external region (MPER) and gp41 glycans [9–17]. BnAbs that target the N332 glycan within the mannose-patch are of particular interest due to their superior breadth and potency and their relatively high frequency in HIV-infected individuals compared to other bnAb specificities [18–20]. BnAbs targeting this region have been isolated from a number of individuals and the prototype bnAbs include 2G12, PGT121, PGT124, 10-1074, PGT128, PGT130 and PGT135 [9–12, 21–24]. BnAb PGT121 has been shown to protect at very low serum concentrations in SHIV challenge studies [7] and to strongly suppress SHIV replication in chronically infected macaques [25].

The PGT125-131 family of bnAbs was isolated from IAVI protocol G donor 36 and have been studied extensively [10, 11, 26, 27]. A crystal structure of PGT128 Fab in complex with the gp120 outer domain showed binding to N-linked glycans at positions N332 and N301 in addition to backbone mediated recognition of ^323^IGDIR^327^ of the V3 loop [10]. We have shown that this family of mannose-patch binding bnAbs can bind promiscuously and use different arrangements of N-linked glycans for neutralization in the absence of the N332 glycan site [27]. More recently we used next generation sequencing and ImmuniTree (a phylogenetic method [28]) to explore the evolution and diversity of the bnAb response in donor 36 and showed that two classes of mannose-patch binding bnAbs exist within this donor [26]. The first and most potent class (prototype PGT128) has a critical 6-amino acid insertion in the CDRH2 and is better able to neutralize viruses with an N332 glycan. The second class (prototype PGT130), although less potent, is more able to tolerate different glycan patterns in the mannose-patch and is more able to neutralize viruses with an N334 glycan [26]. This ability to bind alternate arrangements of glycans in the mannose-patch might limit the ability of the virus to escape through shifting or elimination of glycan sites and led us to explore potential viral escape mechanisms from a promiscuous binding glycan bnAb as found in donor 36.

Recent studies have used bnAbs against the mannose-patch to reduce viral loads in SHIV_SF162P3_ and YU2 chronically infected macaques and humanized mice respectively. In YU2 infected mice, escape from bnAbs 10-1074 and PGT128 occurred through elimination of the N332 glycan site [29]. However, rapid control of virus was observed in these mice when 10-1074 and PGT128 were administered in combination with 3BC176, PG16 and 45-46^G54W^. For SHIV_SF162P3_ infected macaques, PGT121 alone rapidly reduced viral replication but no viral escape was observed [25]. Similarly, Moore et al. have shown, in an HIV-infected individual, that escape from an N332-dependant bnAb response can occur through shifting of the N332 glycan site to the N334 position [30]. Therefore the common assumption has been that escape from mannose-patch binding bnAbs will likely occur through shifting or deletion of the N-linked glycan sites within the glycan shield. However, Sadjadpour et al. recently showed that in vivo escape from an N332-dependant bnAb response in an AD8 infected macaque occurred through a combination of mutations in the V3 loop [31], although these mutations did not confer resistance to donor 36 bnAbs PGT128 or PGT130. Further, Garces et al. showed that PGT124 neutralization resistance could occur through multiple alanine mutations in the ^323^IGDIR^327^ motif in JR-CSF (D325A and R327A) but this also had no impact on PGT128 neutralization [11, 24].

Here we demonstrate the importance of the V3 loop for neutralization and promiscuous binding to the mannose-patch by the donor 36 bnAbs, PGTs125-131. Using an escape virus isolated from donor 36, we show that to restore neutralization sensitivity to the PGT128 bnAb family, mutation to both the V3 glycan positioning and V3 loop protein residues is required and that these mutations can differ for the two bnAb branches. We demonstrate that some of these V3 mutations alone or in combination confer neutralization resistance in other virus strains through both gain-of-function and escape studies. Furthermore, we show that mutation of the V3 can modulate the ability of donor 36 bnAbs to bind promiscuously and use alternative arrangements of N-linked glycans within the mannose-patch. These data highlight the importance of the V3 loop protein epitope for immunogen design strategies aimed at inducing this diverse family of bnAbs through vaccination.

## Results

### CDRH3 interaction with V3 is critical for neutralization sensitivity

**Fig. 1:**
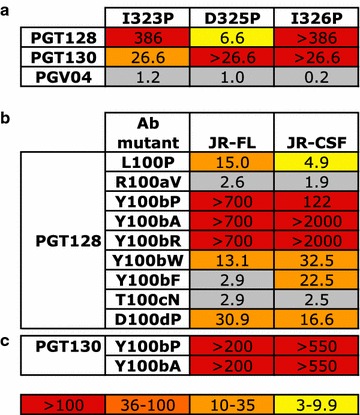
CDRH3 interaction with V3 is critical for neutralization by PGT128 and PGT130. **a** Residues in the ^323^IGDIR^327^ motif of JR-FL were mutated to proline using site-directed mutagenesis and impact on neutralization by PGT128, PGT130 and PGV04 determined. Mutations G324P and R327P produced non-infectious virus. V3 contact residues in the CDRH3 of **b** PGT128 and **c** PGT130 were reverted to germ line or mutated to proline using site-directed mutagenesis and impact on neutralization of JR-FL and JR-CSF determined. Data is reported as fold change in IC_50_ compared to WT (Fold change = IC_50_ mutant/IC_50_ WT) and colour coded according to the figure key

Previous studies had not identified the V3 loop as a potential escape mechanism for PGTs125-131 [11, 24, 31]. It had been suggested that because the V3 contacts with PGT128 CDRH3 are mainly mediated through backbone hydrogen bonding and van der Waals interactions, as observed in the PGT128-eODmV3 crystal structure, that this interaction is tolerant of side-chain variation [10]. However the V3-PGT128 Fab interaction comprises 305 Å^2^ of the 1081 Å^2^ buried surface area suggesting this is an important component of the antibody epitope [10]. To measure the importance of this protein–protein interaction for neutralization and gp120 binding we used proline-scanning mutagenesis of both the V3 loop and CDRH3 loop of PGT128 to distort the β-strand interaction (Fig. 1). Previous alanine-scanning mutagenesis of this region had shown no effect on PGT128 neutralization [11, 24]. First, the ^323^IGDIR^327^ residues of clade B virus JR-FL were mutated to proline, however the G324P and R327P mutations gave non-infectious virus. The other 3 mutations, I323P, D325P and I326P, all reduced the neutralization potency of both PGT128 and PGT130, albeit to varying degrees, but had little impact on PGV04 neutralization (a CD4 binding site bnAb control) (Fig. 1a). Next, mutations in the CDRH3 of PGT128 were made and impact on neutralization determined. Residues within ^100^LRYTD^100d^ were initially either reverted to that of the predicted germ line antibody [26] or, when the residue was unchanged from germ line, mutated to proline. The Y100bP mutation had the largest effect abolishing neutralization of both JR-FL and JR-CSF viruses (Fig. 1b). The same effect was observed for Y100bA and Y100bR mutations. However, when an aromatic residue was substituted at this position (mutants Y100bW and Y100bF) a more modest impact on neutralization by PGT128 was observed highlighting the importance of a hydrophobic interaction with V3 at this position for neutralization. Mutation of PGT130 at position Y100b was also shown to be critical for neutralization (Fig. 1c). Combined, these data demonstrate the importance of the V3-CDRH3 loop interaction for neutralization by this family of bnAbs.

### PGT128 and PGT130 do not neutralize virus isolated from donor 36 at the same time point

Next, to explore possible escape pathways for this family of bnAbs, HIV Env sequences from the time point from which PBMCs were isolated to generate PGTs125-131 were obtained from donor 36 patient sera. Longitudinal samples were not available for this donor and therefore only a single time point could be studied. Unfortunately, only one Env sequence was identified. This was cloned into an Env expression vector for pseudovirus production and used to probe possible escape pathways by this family of bnAbs.

**Fig. 2:**
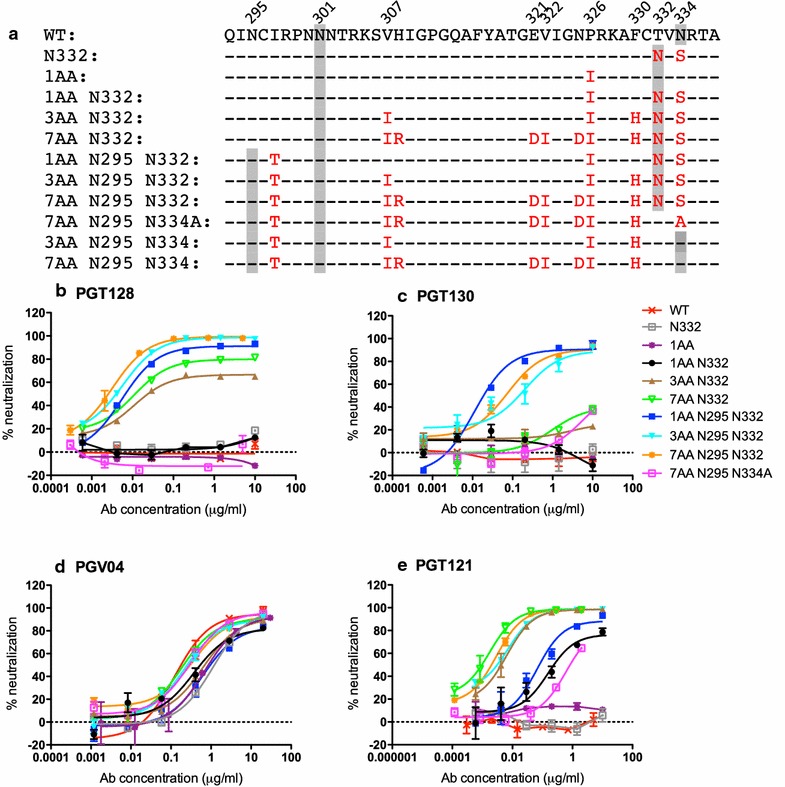
Neutralization of Donor 36 escape virus by HIV bnAbs and representative sequences. **a** Sequence of the V3 region (residues 295–337) from donor 36 virus. Asparagine residues that form part of an N-linked glycosylation sequence are shown in *grey*. Residues that have been mutated from wild-type (WT) are shown in *red*. Neutralization of donor 36 WT and mutated viruses by **b** PGT128, **c** PGT130, **d** PGV04 and **e** PGT121

The virus sequence was subtype CRF02_AG and at the time point PGTs125-131 were isolated had several distinguishing features (Fig. 2a). Firstly, there was a glycan at position 334 rather than the more common 332 position (69.9 % conserved for all HIV Env sequences (Table 1A) and 81.0 % conserved for subtype CRF02_AG (Table 1B), secondly there was an unusual proline residue at position 326 (0.8 % conserved in all HIV Env sequences and 0 % conserved for subtype CRF02_AG), and thirdly there was no glycan at position 295 (50.2 % conserved for HIV Env sequences and 69 % conserved for subtype CRF02_AG). As expected, none of the bnAbs isolated from this donor were able to neutralize this virus (referred to throughout as wild-type, WT) (Fig. 2b, c and Additional file 1: Figure S1). This is consistent with a number of studies showing that, in general, circulating HIV is resistant to the contemporaneous nAbs [32–34]. A control antibody, PGV04 [35], could neutralize the WT virus with an IC_50_ of 0.50 μg/mL (Fig. 2d).

**Table 1:** Conservation of V3 loop residues in HIV Env sequences from the Los Alamos database for A) all clades and B) clade CRF02_AG

A: All clades					
Position	Donor 36	1	2	3	4
295	N	N* (50.2)	I (12.9)	V (12.6)	T (8.8)
301	N*	N* (79.1)	N (10.5)	G (2.1)	T (1.4)
307	V	I (61.4)	V (16.8)	S (8.8)	M (3.7)
308	H	R (34.9)	H (25.1)	I (8.6)	T (6.9)
320	G	G (75.1)	T (8.1)	N (6.2)	E (2.7)
321	E	D (45.0)	E (22.7)	A (7.2)	Q (5.5)
322	V	I (89.4)	V (6.9)	G (1.0)	T (0.4)
325	N	D (78.3)	N (16.9)	I (1.2)	K (0.8)
326	P	I (94.6)	T (1.2)	G (1.0)	P (0.8)
330	F	H (68.9)	Y (25.7)	F (1.8)	Q (1.1)
332	T	N* (69.9)	E (8.5)	T (5.9)	N (4.8)
334	N*	S (69.4)	N* (23.2)	T (2.8)	N (2.2)

B: clade CRF02_AG					
Position	Donor 36	1	2	3	4
295	N	N* (68.6)	T (10.7)	I (9.1)	N (4.1)
301	N*	N* (83.4)	N (5.8)	G (3.3)	K (1.6)
307	V	V (62.8)	I (21.5)	S (6.1)	G (2.5)
308	H	R (64.5)	H (20.7)	V (5.8)	M (1.7)
320	G	G (81.8)	T (6.6)	N (3.3)	D (3.3)
321	E	D (56.2)	E (21.5)	A (7.4)	G (5.0)
322	V	I (90.1)	V (7.4)	T (0.8)	Y (0.8)
325	N	D (71.9)	N (24.0)	G (2.5)	K (1.7)
326	P	I (96.7)	D (1.7)	T (0.8)	K (0.8)
330	F	H (74.4)	Y (21.5)	S (1.7)	Q (0.8)
332	T	N* (81.0)	T (6.6)	N (4.1)	K (1.7)
334	N*	S (81.8)	N* (14.9)	T (1.7)	V (0.8)

Values are based on 4907 and 121 sequences from the Los Alamos database for all clades and clade CRF02_AG respectively. Values are reported as  % occurrence and only the four most frequent residues are shown. N* refers to asparagine residues that are part of a glycosylation sequon and N refers to asparagine residues that are not

### Mutations in glycan positioning and V3 loop protein residues confer neutralization sensitivity for PGT128 and PGT130

We next used site-directed mutagenesis to introduce mutations into Env in an attempt to confer neutralization sensitivity to the donor 36 bnAbs. We focused initially on the V3 loop region and N-linked glycan sites within (N295, N301 and N332 or N334) as these had previously been shown to be important for neutralization by the donor 36 bnAbs [10, 11, 27]. These residues were mutated toward consensus of subtype CRF02_AG viruses (Table 1) and the most broad and potent bnAbs from the two branches, PGT128 and PGT130, along with PGT121, a mannose-patch binding bnAb from another Protocol G donor [11, 28], and the CD4 binding site bnAb PGV04 [35] were tested. Mutations were introduced both alone and in combination and are summarized in Fig. 2a. Substitutions that shifted the glycan site from position 334 to 332 alone were insufficient to confer neutralization sensitivity to PGT128, PGT130 or PGT121 (Fig. 2b, c, e). Similarly, mutation of P326 to the more commonly observed isoleucine (referred to as 1AA virus) alone or in combination with the N334–N332 shift was insufficient to generate neutralization sensitivity to PGT128 and PGT130 (Fig. 2b, c). However, PGT121 neutralized virus 1AA N332 potently (Fig. 2e) highlighting the differences in V3 dependence between mannose-patch binding bnAb families. The neutralization potency of PGV04 was mostly unaffected by the mutations made (Fig. 2d).

Alignment of sequences from the Los Alamos database allowed additional residues to be identified within the V3 loop that were less conserved compared to known subtype CRF02_AG viruses. A variant with a further two mutations V307I and F330H, which are 16.8 % (V) and 1.8 % (F) conserved across all HIV sequences respectively and 63 % (V) and 0 % (F) conserved among CFR02_AG viruses respectively (Table 1) (referred to as 3AA N332 virus), was neutralized by PGT128. However, the neutralization curve plateaued at around 75 % (Fig. 2b) and this virus remained resistant to PGT130 neutralization (Fig. 2c). The low plateau of neutralization suggested that there was a population of viruses that were still resistant to PGT128 neutralization [26, 36, 37]. We therefore looked to introduce additional protein and glycan site mutations that might increase the neutralization plateau to 100 %. Addition of a further four mutations, H308R, E321D, V322I and N325D (referred to as 7AA N332 virus), resulted in a slightly increased neutralization plateau of 85 % for PGT128, and PGT130 now neutralized to approximately 40 % at 10 μg/mL (Fig. 2b, c). The continued importance of the original four mutations for neutralization sensitivity in the 7AA N332 virus was confirmed by reverting I307, I326, H330 and N332 of 7AA N332 individually back to WT (Additional file 1: Figure S2).

As mentioned above, the WT virus did not have a glycan at position N295. When this glycan was introduced (using an I297T mutation) into the 1AA, 3AA and 7AA N332 variants both PGT128 and PGT130 were able to neutralize all viruses with a plateau of >90 % (Fig. 2b, c). However, the presence of the N295 glycan in the absence of N332 (7AA N295 N334A) was insufficient for neutralization (Fig. 2b, c). Similar neutralization patterns were observed for the remaining bnAb family members; PGT125, PGT126, PGT127 and PGT131 (Additional file 1: Figure S1). These results show that mutation in both the glycan shield and the V3 loop core epitope are required to restore neutralization sensitivity to the escape virus studied.

### PGT128 and PGT130 bnAb branches show different dependencies on V3 glycan site positioning in donor 36 virus

**Fig. 3:**
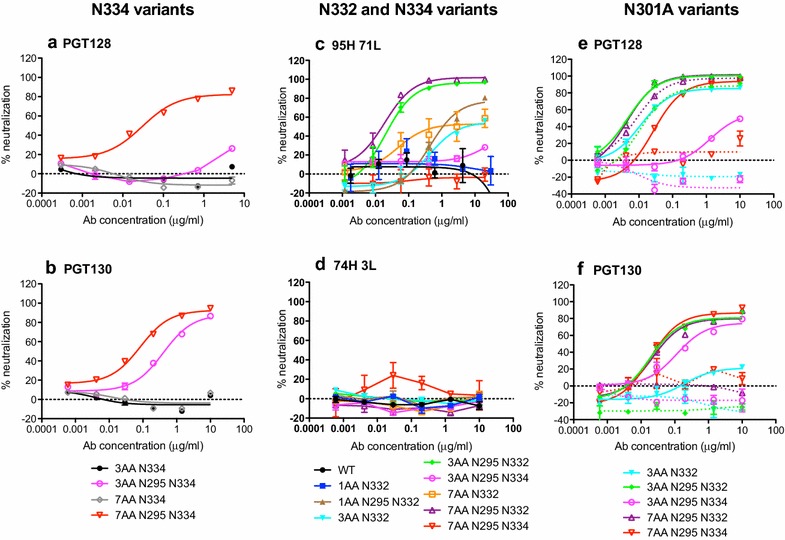
PGT128 and PGT130 show different dependencies on V3 loop N-linked glycosylation sites. Neutralization of donor 36 viruses with an N334 glycan site by bnAbs **a** PGT128 and **b** PGT130. Neutralization of donor 36 viruses with an N332 or N334 glycan site by Abs **c** 95H 71L (less mutated variant of PGT128, 12 % mutated in V_H_ and V_L_) and **d** 74H 3L (less mutated variant of PGT130, 13 % mutated in V_H_ and V_L_). Importance of N301 glycan in the donor 36 virus for neutralization by bnAbs **e** PGT128 and **f** PGT130. The Asn at position N301 was mutated to alanine in the 3AA N332, 3AA N295 N332, 3AA N295 N334, 7AA N295 N332 and 7AA N295 N334 variant viruses using site-directed mutagenesis and impact on neutralization measured. The N301A variants are shown with the *dotted line*

We have recently used next generation sequencing and ImmuniTree [28] to explore the evolution of the bnAb response in donor 36 and to predict less mutated bnAbs [26]. We showed that PGT128 and 95H 71L (a less mutated form of PGT128) preferentially neutralized N332 viruses whereas PGT130 and 74H 3L (a less mutated form of PGT130) preferentially neutralized N334 virus [26, 27]. To determine whether differing sensitivities of PGT128, 95H 71L, PGT130 and 74H 3L towards the N332 and N334 versions of the infecting viruses may have contributed to divergence in epitope recognition in this donor we generated N332 and N334 versions of the 3AA and 7AA viruses both with and without the N295 and/or the N301 glycans present (Fig. 3).

Firstly, we investigated the impact of the presence of the N295 glycan in the different N334 virus variants. PGT128 could only neutralize the N334 variant when the N295 glycan and all V3 mutations were also present (i.e. 7AA N334 N295 variant, Fig. 3a). In contrast, PGT130 was able to neutralize both the 3AA and 7AA N334 variants, although again only when the N295 glycan was also present (Fig. 3b). Of the less mutated bnAbs, only 95H 71L showed any activity, neutralizing only the N332 mutant viruses, albeit with a greatly reduced neutralization plateau (60 % compared to 80 % for PGT128), and the N295/N332 mutant viruses with plateaus of almost 100 % (Fig. 3c). 74H 3L was not able to neutralize any of the viruses tested (Fig. 3d).

Secondly, we looked at the impact of the glycan at N301 in N332 and N334 viruses. Interestingly, when the N301 glycan was removed from the 3AA N332, 3AA N295 N332, 7AA N295 N334 and 7AA N295 N332 variant viruses, PGT128 was only dependent on N301 for neutralization of the 7AA N295 N334 virus (Fig. 3e), whereas PGT130 was found to be dependent on N301 for all viruses (Fig. 3f) [10, 11]. These data further highlight different dependencies on N-linked glycosylation sites by the two-bnAb classes. Overall, in agreement with our previous observations [26, 27], PGT130 is better able to neutralize the N334 variant donor viruses compared to PGT128, but requires the N295 and N301 glycans to do so.

To investigate whether PGT128 and PGT130 were able to bind the Env of neutralization resistant viruses we performed ELISA assays with captured gp120 from lysed pseudoviruses. The pattern of PGT128 binding the Env mutants was consistent with that seen for neutralization (Additional file 1: Figure S3, and Figs. 2b and 3a). However, PGT130 bound poorly to some gp120s when neutralization of the corresponding virus was potent, e.g. 7AA N295 N334 (Additional file 1: Figure S3, and Figs. 2c and 3b). This is consistent with observations that PGT130 binds poorly to some monomeric gp120s (unpublished data).

### Addition of V1 loop glycans as a potential escape strategy

**Fig. 4:**
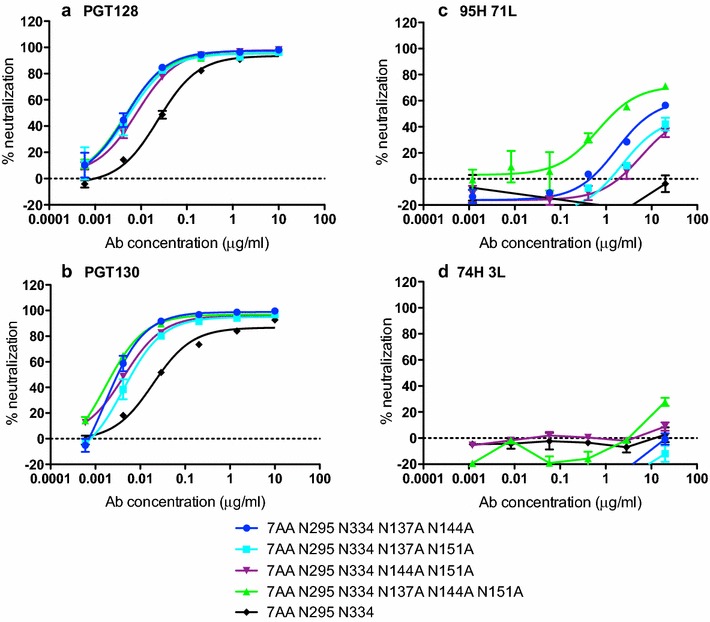
V1 loop glycan addition as a potential escape strategy. V1 loop glycans were removed using an Asn to Ala substitution. Single (see Additional file 1: Figure S4A), double and triple V1 loop glycan mutants were prepared for the 7AA N295 N334 variant. Neutralization was determined for **a** PGT128, **b** PGT130, **c** 95H 71L and **d** 74H 3L

We have previously observed that removal of V1 loop glycan sites can increase neutralization sensitivity of certain less mutated nAbs [26] and this has also been observed for the PGT121 bnAb family [24, 38]. To investigate whether V1 loop glycan addition could be a potential escape strategy in the donor 36 virus, we removed the three V1 loop glycans that were present in the isolated virus sequence (N137, N144 and N151), alone and in combination, from the 7AA N332 and 7AA N295 N334 variants and measured the impact on neutralization sensitivity of PGT128, PGT130, 95H 71L and 74H 3L (Fig. 4a–d and Additional file 1: Figure S4). Due to the preference for N334 variant viruses, 74H 3L was only tested on the 7AA N295 N334 variant [26]. Unlike observations for some other viruses [26], removal of V1 loop glycans individually did not confer neutralization sensitivity to 74H 3L (Additional file 1: Figure S4A), nor did it greatly increase neutralization sensitivity to 95H 71L, PGT128 or PGT130 (Additional file 1: Figure S4A). When these mutations were added in pairs to 7AA N295 N334, an enhancement in neutralization plateau and potency for PGT128 and PGT130 was observed (Fig. 4a, b), and 95H 71L was now able to neutralize this virus (Fig. 4c). Introduction of all three mutations simultaneously further increased the neutralization potency of 95H 71L (Fig. 4c) although 74H 3L remained unable to neutralize the virus (Fig. 4d). Removal of V1 loop glycans in the 7AA N332 variant virus had limited impact on neutralization potency of PGT128 and 95H 71L (Figure S4B). To confirm that the V1 glycan site deletion mutants were not leading to a general increase in neutralization sensitivity, neutralization by a wider panel of HIV bnAbs (PG9, PGT151, PGT145 and PGT135) and HIVIG (pooled IgG from HIV infected individuals) were tested against 7AA N295 N334 and V1 deleted mutant (Additional file 1: Figure S5). No enhancement of neutralization was observed suggesting the effect observed is specific for this bnAb family. These data may indicate addition of V1 loop glycans as a potential escape mechanism from early nAbs for the donor 36 virus, although longitudinal virus sequences would be required to confirm this. The importance of V1 loop glycans for bnAb evolution has also been suggested for the PGT121 family where addition of a glycan at position N137 is thought to drive maturation of the bnAb response [38].

### V3 protein residues facilitate escape in other viruses to differing extents

**Fig. 5:**
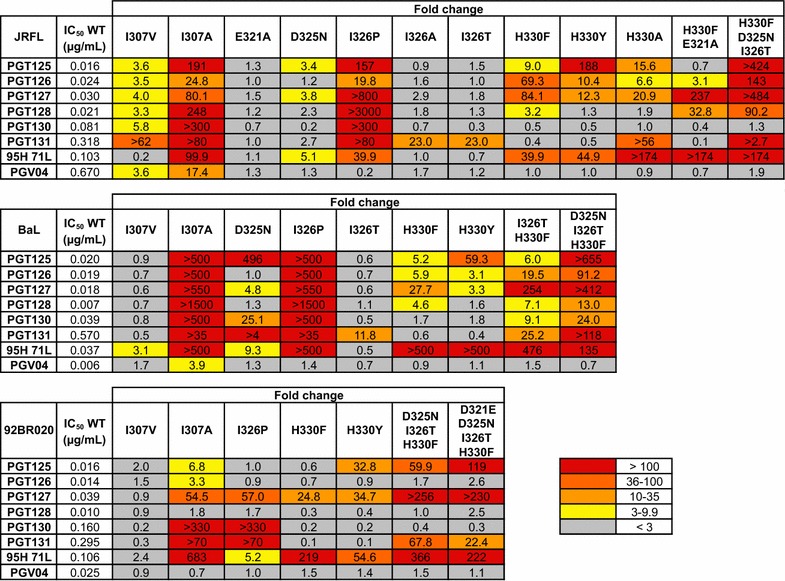
Importance of V3 loop residues at positions 307, 321, 325, 326 and 330 for neutralization by the donor 36 bnAb family. Mutations shown to confer neutralization sensitivity to the donor 36 virus were introduced alone or in combination into BaL, JR-FL and 92BR020 pseudoviruses and the impact on neutralization determined for PGTs125-131, 95H 71L and PGV04. Data is reported as fold change in IC_50_ compared to WT (Fold change = IC_50_ mutant/IC_50_ WT) and colour coded according to the figure key. IC_50_ values for each antibody against the wild-type virus is shown for reference (in µg/ml)

As mentioned above, previous studies have not reported V3 mutations that lead to neutralization resistance by PGT128 [11, 24, 29, 31]. To determine whether the V3 mutations required to restore neutralization sensitivity to the donor 36 virus could facilitate neutralization resistance in the context of other viruses, we next prepared a panel of mutant viruses harbouring these mutations singly and in combination. Mutations were focussed at positions 307, 321, 325, 326 and 330 in the clade B viruses, BaL, JR-FL and 92BR020. The impact on neutralization sensitivity by the full PGT128 bnAb family was measured (Fig. 5).

Both I307A and I307V were tested to maximise the chances of seeing a neutralization effect. The I307A mutation reduced neutralization potency for the majority of the bnAbs and in some cases no neutralization was observed at concentrations of up to 20 μg/mL (Fig. 5). The I307V mutation had only a very modest effect on neutralization potency for JR-FL suggesting that individual subtle changes at this position can be tolerated. The I326P mutation reduced neutralization potency for the majority of bnAbs, although the effect was less dramatic for 92BR020. However, mutation at the 326 position to Thr (2nd most commonly observed in the Los Alamos database (Table 1) or to Ala had no effect on neutralization sensitivity of JR-FL. Mutations H330Y and H330F were both tested at position 330 as these residues are most commonly found in viruses containing a glycan at position N334 rather than position N332. Both mutations had similar but less pronounced effects compared to the I307A and I326P mutations and showed a reduction of neutralization potency and/or neutralization plateau for some bnAbs but never abolished neutralization completely (data not shown). An H330A mutation in JR-FL also had a similar impact on neutralization. Mutation at position 321 and 325 alone (D321A and D325N) had no effect on neutralization. Antibody 95H 71L was much more sensitive to the V3 mutations with larger increases in IC_50_ observed and/or bigger reductions in neutralization plateau compared to PGT128 suggesting increased somatic hypermutation is required for tolerating diversity in V3. Control bnAb PGV04 was largely unaffected by the mutations made.

To investigate whether combinations of these mutations could facilitate escape, we prepared several double, triple and quadruple mutants in all three-virus strains. In general, double mutations had limited additional impact on neutralization potency compared to single mutations. For JR-FL and BaL, only the D325N + I326T + H330F triple mutant was prepared as both viruses already had a D at position 321. For both triple mutants, neutralization was completely abolished for PGT125, PGT127 and PGT131 and neutralization potency was reduced for PGT126, PGT128 and PGT130. Similarly, for the 92BR020 quadruple mutant, PGT125, PGT127 and PGT131 had greatly decreased neutralization potency but this effect was minimal for PGT126, PGT128 and PGT130. In all cases 95H 71L was more sensitive to the V3 mutations. Overall, these results highlight the importance of the V3 loop for neutralization efficiency and as a potential viral escape mechanism. Although individual mutations had limited effect on neutralization, these effects were enhanced when mutations were combined as previously observed for the PGT121 bnAb family [24, 31].

### Residues in V3 can modulate promiscuous binding to the mannose-patch

**Fig. 6:**
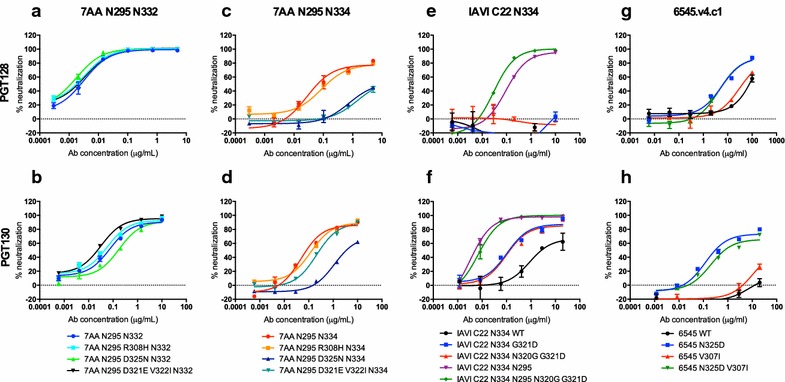
Importance of V3 loop residues for promiscuous binding of the mannose-patch. V3 loop residues in the **a**, **b** 7AA N295 N332 and **c**, **d** 7AA N295 N334 variants were mutated to WT and the impact on neutralization measured for PGT128 and PGT130. Mutations in the V3 loop were made to **e**, **f** IAVI C22 N334 and **g**, **h** 6545.v4.c1 to confer neutralization sensitivity by PGT128 and PGT130

The difference in sensitivity of the 3AA N295 N334 and 7AA N295 N334 viruses to neutralization by PGT128 (Fig. 3a) suggests that the ability of the antibody to bind promiscuously to the mannose-patch is somewhat dependent upon protein residues in the V3 loop. To test this hypothesis we reverted the additional V3 residues R308, D321, V322 and D325 back to WT for both the N332 and N334 versions of the 7AA N295 virus and measured the impact on neutralization (Fig. 6). For the 7AA N295 N332 variant these mutations had little or no effect on neutralization for both PGT128 and PGT130 (Fig. 6a, b). However, for the 7AA N295 N334 variant, the potency of PGT128 neutralization was reduced by the D325N and D321E V332I mutations and PGT130 neutralization was reduced by the D325N mutation (Fig. 6c, d). As the I323 and D325 residues have been shown to contact the CDRH3 of PGT128 via the protein backbone, these findings suggest that certain residues present in the V3 protein contact region may be required to help position the N334 glycan such that PGT128 and PGT130 can bind and this is more apparent for PGT128. Alternatively, some side chain binding with the ^323^IGDIR^327^ motif may occur when the N334 glycan site is present that is not observed in the N332 virus. Either way, these data highlight the importance of V3 residues in promiscuous binding of donor 36 bnAbs to the mannose-patch.

To further investigate the importance of the V3 loop for promiscuous glycan binding, we introduced V3 mutations into N334 and N332A resistant viruses in an attempt to restore PGT128 and PGT130 neutralization sensitivity (Fig. 6e–h). We have previously shown that PGT128 is unable to neutralize the N332A and N334 variants of clade C virus IAVI C22 and PGT130 has severely decreased neutralization potency compared to WT [26, 27]. Inspection of the sequence revealed that this virus lacked a glycan at N295 and had Asn at position 320 and Gly at position 321 instead of the more common Gly and Asp respectively (Table 1). The N320G and G321D mutations were introduced and the N295 glycan site added (through a V295N mutation) alone and in combination to the N332A and N334 IAVI C22 variants and impact on PGTs125–131, 95H 71L and PGV04 neutralization determined (Fig. 6e, f, Additional file 1: Figure S6A, B). Neutralization by PGT128 could only be restored by introduction of the N295 glycan (Fig. 6e and Additional file 1: Figure S6A, B), which is consistent with the use of this glycan in the absence of N332 [10, 27]. However, neutralization of PGT130 could be enhanced through V3 mutation G321D alone and this was further enhanced by addition of the N295 glycan (Fig. 6f and Additional file 1: Figure S6A, B). The less mutated bnAb 95H 71L remained resistant to all viruses and PGV04 was unaffected (Additional file 1: Figure S6A, B).

We next identified two viruses that naturally have N334 and N295 glycan sites but are largely resistant to neutralization by PGTs125-131 and attempted to restore neutralization through mutation of the V3 protein residues alone [27]. An E321D mutation was introduced into the clade B virus REJO4541.67 but the mutant virus remained resistant to neutralization by PGTs125-131 (data not shown). However, when an N325D mutation was introduced into clade AC virus 6545.v4.c1, PGT125, PGT128, PGT130 and PGT131 were able to neutralize the mutant virus (Fig. 6g, h and Additional file 1: Figure S6C) and neutralization potency could be further increased through addition of the V307I mutation. Combined, these data highlight the importance of the V3 loop for modulating promiscuous binding of the mannose-patch by this family of bnAbs and likely represent an alternative escape pathway to glycan site deletion.

## Discussion

We have previously shown that PGTs125-131 are promiscuous in their recognition of the mannose-patch and are able to neutralize a considerable proportion of viruses either lacking the N332 glycan site or with the glycan site shifted to the 334 position [27]. This led us to explore viral escape mechanisms from this promiscuous glycan-binding bnAb family using an escape virus isolated from donor 36 as a starting point. We describe three observations that have implications for HIV-1 vaccine design. Firstly, different mutations in the virus isolated from donor 36 can restore neutralization sensitivity towards bnAbs PGT128 and PGT130, secondly mutations in both the protein sequence and glycan positions can lead to neutralization resistance, and thirdly, the V3 loop protein residues can play an important role in modulating promiscuous binding to the mannose-patch.

**Fig. 7:**
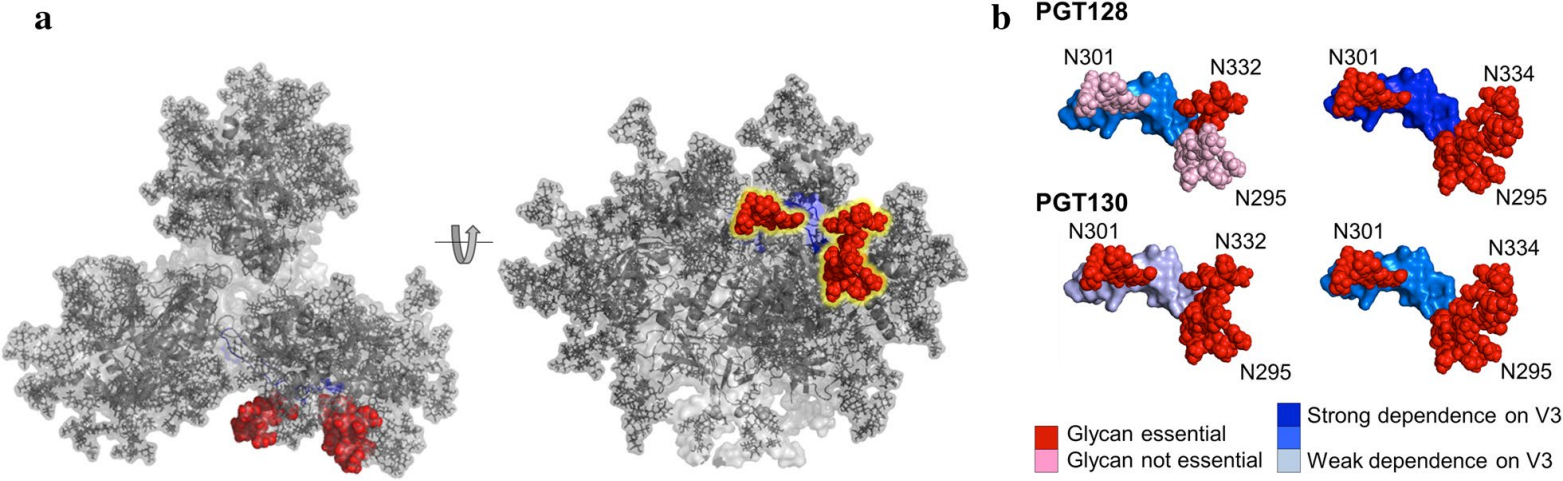
Model depicting the differences in dependency on the V3 loop and surrounding glycan sites (N295, N301, N332/N334) for PGT128 and PGT130 based on the donor 36 virus. **a** Model of bnAb epitope on the HIV trimer. **b** PGT128 neutralizes the N332 viruses very potently and requires glycosylation at either position N295 or N301. However, PGT128 can only neutralize the N334 variant when both glycan sites at N295 and N301 are present. PGT130 on the other hand neutralizes both N332 and N334 variants very potently, but depends on the presence of both glycan sites at N295 and N301. Differences are also observed in the V3 loop residues that should be reverted to confer and improve neutralization. PGT128 is less dependent on the V3 loop composition when N332 is glycosylated. It neutralizes the 1AA mutant virus potently and with increasing changes in V3, its potency, as well as the neutralization plateau, increases. If the glycan is at position N334, the bnAb only neutralizes the 7AA variant. The neutralization potency of PGT130, however, decreases slightly with increasing changes in the V3 loop in the N332 virus variants, hinting that PGT130 is less dependent on V3 loop conformation. In the presence of the N334 glycan, PGT130 only neutralized the 3AA and 7AA mutants and potency, as well as the neutralization plateau, increases when more changes were introduced. The dependencies are highlighted in accordance with the figure key

We have previously shown that the bnAb response in donor 36 is diverse such that two classes of bnAb exist [26]. Figure 7 models the different dependencies on V3 and surrounding glycans for the PGT128 and PGT130 bnAb classes based on the isolated donor 36 virus. For a virus displaying an N332 glycan, PGT128 requires either the N295 or N301 glycan for neutralization and additional mutations in V3 increase the neutralization plateau (Fig. 7b). However, for a virus displaying an N334 glycan, PGT128 requires both N295 and N301 glycans and is more sensitive to mutations in the V3 loop, only neutralizing the 7AA variant. PGT130 differs in that neutralization of both N332 and N334 containing viruses requires both the N295 and N301 glycans (Fig. 7b). PGT130 dependency on V3 loop mutations for neutralization is greatest for N334 viruses although, unlike PGT128, it is able to neutralize both the 3AA and 7AA versions. Neutralization sensitivity towards the donor 36 virus can be conferred for both bnAb classes through addition of the N295 glycan site, P326I mutation and shifting of the N334 glycan site to N332. However, neutralization by PGT128 can also be conferred with the 3AA N332 mutant and for PGT130, it can be conferred through the 3AA N295 mutant. These differences in sensitivity towards the host virus might have contributed towards divergence of recognition between the two bnAb classes (although longitudinal Env sequencing would be required to determine this).

We next consider the role of the V3 loop in neutralization resistance and modulating promiscuous binding of the mannose-patch. Neutralization escape via protein mutation in V3 has not previously been reported for PGTs125-131 with the commonly reported escape through an N332A or N301A mutation [10, 11, 27]. For viruses where an N332A mutation is insufficient for neutralization resistance, a double glycan site deletion (e.g. N332A + N295A) can abolish neutralization [27]. Here we extend these observations and demonstrate that either combined mutations at positions 321, 325, 326 and 330 or glycan site deletion/shifting combined with V3 mutation can also facilitate viral escape. There are limited studies determining escape from an N332-bnAb response in vivo. Sadjadpour et al. reported escape from an N332-dependent bnAb response in an SHIV_AD8_ infected macaque mainly occurred through stepwise substitution of amino acids in the C-terminal end of the V3 loop [31]. A minimum of two mutations (D321A and I323T) was required for escape from the serum antibodies (although these mutations did not confer resistance to PGT128 or PGT130). In contrast, Moore et al. reported escape in two chronically infected individuals occurred through shifting of the N332 glycan site to N334 [30]. Accompanying mutations at positions 325 and 330 were observed in some escape viruses although the importance of these mutations was not determined. Interestingly, despite the prevalence of mannose-patch binding bnAbs within HIV-1 infected individuals [18], 90 % of viruses have a glycan at N332 or N334 [27]. This may indicate an unrealized selection pressure for Env to maintain the N332/N334 glycan site for Env stability or viral fitness. Further longitudinal analysis of viral sequences from N332A sensitive HIV donors may reveal a preference for escape via V3 rather than glycan-site deletion or shifting.

Neutralization resistance via mutation in V3 is emerging as a common feature for mannose-patch binding bnAb families, e.g. PGT121 and PGT135 bnAb families [9, 24]. However, as mentioned above, different mutations facilitate resistance for the different antibody lineages [9, 24, 31] and this is further exemplified here by the different mutations required to confer neutralization sensitivity against the donor 36 virus by PGT121 and PGT128/130. These differences in V3 escape for N332-dependent bnAb lineages further highlight the diverse mechanisms used by bnAbs to bind the N332 ‘supersite of vulnerability’ [9] and further support the idea that eliciting a diverse bnAb response against this epitope may minimize viral escape and increase the breadth of protection [27].

It is not clear how protein residues in the V3 loop modulate the ability of bnAbs to bind alternative arrangements of glycans in the mannose-patch. A recent EM structure of PGT128 Fab in complex with BG505 SOSIP.664 trimer indicates that the N295 glycan would have to move approximately 60 Å to interact with PGT128 in the absence of N332 [39]. However, a recent crystal structure of PGT128 Fab in complex with BG505 SOSIP.664 trimer shows the D1 arms of N295 and N332 are separated by only 5 Å suggesting a minor shift would facilitate binding to N295 in the absence of N332 [40]. Residues in V3 may help position the N334 or N295 glycan to enable simultaneous binding of the N334/N295 and N301 glycan sites and V3 loop. Alternatively, when the N334 glycan is present interaction with V3 may be more dependent on side-chain than backbone interactions and therefore bnAbs can tolerate less variation in V3 for an N334 virus. Additional structural studies of PGT128 and PGT130 Fab with Env proteins harbouring an N334 glycan site would provide greater insight into how the V3 mutations impact the epitope recognition by this family of bnAbs.

These observations have implications for immunogen design. As the most efficacious vaccine response would likely include both the PGT128 and PGT130 bnAb classes [26, 27], immunogens displaying different arrangements of glycans in the V3 region may be required to elicit a diverse bnAb response as observed in donor 36. We suggest a priming immunogen should include the most conserved V3 residues and the N295 and N332 glycan sites but have fewer V1 loop glycans to enhance epitope recognition. Sequential booster immunogens should have shifted glycan sites and include N295/N334, N332 alone or N334 alone, although it is not clear from this data which order would be most optimal and in vivo models will be required to determine this. Our results also highlight the importance of V3 for immunogen design aimed at inducing bnAbs that can bind promiscuously to the mannose-patch. Current immunogen design strategies commonly incorporate a truncated ‘mini’ V3 [41]. However these data suggest a full V3 loop is likely required to generate bnAbs that can tolerate the inherent level of variability in V3. Therefore, some variation in the V3 region should be incorporated into these glycan-shifted immunogens to help generate bnAbs that can tolerate mutation in the V3 region. This is supported by the observation that the less mutated bnAb, 95H 71L, is more sensitive to protein changes in the V3 region. As the V3 requirements appear to differ for the different bnAb lineages that recognize this region of Env, different immunogens may be optimal for eliciting certain bnAb lineages.

## Conclusions

In summary, we show that neutralization resistance from this bnAb family can occur via mutation of protein residues in the V3 region, as well as shifting and/or deletion of N-linked glycans. We show that residues in V3 can have an important impact on the ability of donor 36 bnAbs to bind promiscuously to the mannose-patch. We further show that different mutations in the escape virus isolated from donor 36 can restore neutralization sensitivity towards the two bnAb branches. These differences may have led to divergence in mannose-patch recognition (although longitudinal analysis would be required to prove this). These observations highlight the importance of the V3 loop for design of immunogens capable of eliciting Abs similar to the diverse family of bnAbs described here.

## Methods

### Ethics statement

Plasma was obtained from donor 36, an HIV-1 infected donor from the IAVI Protocol G cohort [42]. All human samples in Protocol G were collected with written informed consent under clinical protocols approved by the Republic of Rwanda National Ethics Committee, the Emory University Institutional Review Board, the University of Zambia Research Ethics Committee, the Charing Cross Research Ethics Committee, the UVRI Science and Ethics Committee, the University of New South Wales Research Ethics Committee. St. Vincent’s Hospital and Eastern Sydney Area Health Service, Kenyatta National Hospital Ethics and Research Committee, University of Cape Town Research Ethics Committee, the International Institutional Review Board, the Mahidol University Ethics Committee, the Walter Reed Army Institute of Research (WRAIR) Institutional Review Board, and the Ivory Coast Comité National d’Ethique des Sciences de la Vie et de la Santé (CNESVS).

### Amplification and expression of donor HIV-1 gp160 sequences

HIV Env sequence was determined as previously described [43]. Briefly, virus was pelleted by centrifugation of patient plasma (20,400 g, 1 h). Viral particles were lysed by resuspension in lysis buffer (4 M guanidine thiocyanate, 0.1 M Tris HCl, pH 8.0, 0.5 % sodium lauryl sarcosine, and 1 % dithiothreitol). RNA was extracted using oligo(dT) linked to magnetic beads (Dynal, Oslo, Norway) and cDNA was synthesised in a standard RT reaction using oligo(dT) primers and Thermoscript RT enzyme (Invitrogen, Gaithersburg, MD). Env regions were amplified with BD Advantage 2 PCR Enzyme System (Clontech, Mountain View, CA) using a forward and reverse primer containing the *PinAI* and *MluI* restriction sites respectively. PCR products were digested with *PinAI* and *MluI*, purified by agarose gel electrophoresis and ligated to *PinA1* and *MluI*-digested envelope expression test vector (eETV). Ligation reactions were used to transform competent *E. coli* and the expression plasmids were purified using silica column chromatography (Qiagen, Valencia, CA). Colonies were picked and screened by a rapid single replication cycle assay. One viable viral clone was identified and selected for sequencing and further testing.

The Env sequence was synthesised by GENEWIZ (South Plainfield, NJ) and cloned into the pSVIII vector using the KpnI and XhoI restriction sites for use in pseudovirus assays.

### Envelope and antibody mutations

Mutations in the HIV-1 envelope glycoproteins or antibody expression plasmids were introduced using QuikChange site-directed mutagenesis (Stratagene, La Jolla, CA). Mutations were verified by DNA sequencing (Eton Biosciences, La Jolla, CA and MWG Eurofins, Germany).

### Pseudovirus production and neutralization assays

To produce pseudoviruses, plasmids encoding Env were co-transfected with an Env-deficient genomic backbone plasmid (pSG3∆Env) in a 1:2 ratio with the transfection reagent PEI (1 mg/mL, 1:3 PEI:total DNA, Polysciences) into HEK 293T cells [44, 45]. Pseudoviruses were harvested 72 h post transfection for use in neutralization assays.

Neutralizing activity was assessed using a single round replication pseudovirus assay with TZM-bl target cells, as described previously [44, 45]. Briefly, the antibody or HIVIG (HIV hyperimmune globulin) was serial diluted in a 96 well flat bottom plate and pre-incubated with virus for 1 h at 37 °C. Cells at a concentration of 20,000 cells/well (supplemented with 10 μg/ml DEAE-Dextran for all donor virus variants) were added to the virus/antibody mixture and luminescence was quantified 72 h following infection via lysis and addition of Bright-Glo™ Luciferase substrate (Promega). Dose–response curves were fitted using nonlinear regression (GraphPad Prism) to determine IC_50_ values.

### Gp120 ELISA

For binding to gp120 isolated from pseudovirus, virus was collected 3 days post transfection, supernatants were spun down at 300 g for 5 min, and virus was lysed with 1 % NP-40 at room temperature (RT) for 30 min. Enzyme-linked immunosorbent assay (ELISA) plates were coated overnight at 4 °C with anti-gp120 Ab D7324 (Aalto Bio Reagents, Dublin) at a concentration of 3 μg/ml in PBS. Plates were washed 4 times with PBS-T (PBS with 0.05 % Tween-20) and blocked for 1 h with 100 μl 5 % non-fat milk in PBS-T. Lysed virus was added at 50 μl/well and incubated for 2 h and washed 4 times with PBS-T. Serial dilutions of bnAb in 5 % nonfat milk/PBS-T were incubated for 2 h. Unbound antibody was removed by washing four times as described above. 50 μl of biotin conjugated rabbit anti-human Fc antibody was added (1:1000), incubated for 30 min and washed 4 times. Binding was detected with streptavidin–alkaline phosphatase conjugate (1:1000; BioRad), and *p*-nitrophenol phosphate substrate (Sigma) at 405 nm.

### Antibody expression and purification

Antibody plasmids were co-transfected at a 1:1 ratio in 293 freestyle cells using 293fectin (Invitrogen) or with the transfection reagent PEI (1 mg/mL, 1:3 PEI:total DNA, Polysciences). Transfections were performed according to the manufacturer’s protocol and antibody supernatants were harvested 4 days following transfection. Antibody supernatants were purified over a protein A column, eluted with 0.1 M Glycine (pH 3.5), and buffer exchanged into PBS.

## Additional file

10.1186/s12977-016-0241-5 **Figure S1.** Neutralization of donor 36 WT and mutated viruses by the remaining donor 36 bnAbs. **A** PGT125, **B** PGT126, **C** PGT127, and **D** PGT131. **Figure S2.** Importance of I307, I326, Y330 and N332 for neutralization of 7AA N332 variant virus. Residues at positions 307, 326, 330 and 332 were reverted to WT and the impact on neutralization measured for **A** PGT128, **B** PGT130 and **C** PGV04. **Figure S3.** Binding to Env variants in ELISA. Gp120 from lysed pseudovirions was captured using an anti-gp120 antibody (Ab D7324, Aalto Bio Reagents, Dublin) and binding by a **A** PGT128, **B** PGT130 and **C** PGV04 measured. Binding to gp120 closely matched neutralization for PGT128 however binding by PGT130 was reduced. **Figure S4.** Dependency on V1 loop glycans for neutralization by donor 36 bnAbs. **A** Neutralization of 7AA N295 N334 virus variants lacking individual V1 loop glycans. **B** Neutralization of 7AA N332 virus variants lacking individual V1 loop glycans. **Figure S5.** Dependency on V1 loop glycans for neutralization by a panel of HIV bnAbs and HIVIG. Neutralization of 7AA N295 N334 virus variants lacking all three V1 loop glycans. **A** PGT135, **B** PG9, **C** PGT145, **D** PGT151, **E** HIVIG (HIV hyperimmune globulin) and **F** PGV04. **Figure S6.** Residues in V3 are important for promiscuous binding of the mannose-patch. V3 residues in **A** IAVI C22 N332A, **B** IAVI C22 N334 and **C** 6545.v4.c1 were mutated to confer neutralization by the PGTs125-131. Numbers represent IC_50_ values for each antibody and are reported as µg/ml.

## Abbreviations

HIV-1: Human Immunodeficiency Virus-1
bnAb: broadly neutralizing antibody
Env: envelope
IAVI: International AIDS Vaccine Initiative
MPER: membrane proximal external region
WT: wild-type
