# Supplementary material for: Mechanisms of escape from the PGT128 family of anti-HIV broadly neutralizing antibodies
Source: Retrovirology. 2016 Feb 2;13:8. doi: 10.1186/s12977-016-0241-5 (PMC4736637; doi:10.1186/s12977-016-0241-5)
Supplement: Supplementary file 1 — 10.1186/s12977-016-0241-5 Figure S1. Neutralization of donor 36 WT and mutated viruses by the remaining donor 36 bnAbs. A PGT125, B PGT126, C PGT127, and D PGT131. Figure S2. Importance of I307, I326, Y330 and N332 for neutralization of 7AA N332 variant virus. Residues at positions 307, 326, 330 and 332 were reverted to WT and the impact on neutralization measured for A PGT128, B PGT130 and C PGV04. Figure S3. Binding to Env variants in ELISA. Gp120 from lysed pseudovirions was captured using an anti-gp120 antibody (Ab D7324, Aalto Bio Reagents, Dublin) and binding by a A PGT128, B PGT130 and C PGV04 measured. Binding to gp120 closely matched neutralization for PGT128 however binding by PGT130 was reduced. Figure S4. Dependency on V1 loop glycans for neutralization by donor 36 bnAbs. A Neutralization of 7AA N295 N334 virus variants lacking individual V1 loop glycans. B Neutralization of 7AA N332 virus variants lacking individual V1 loop glycans. Figure S5. Dependency on V1 loop glycans for neutralization by a panel of HIV bnAbs and HIVIG. Neutralization of 7AA N295 N334 virus variants lacking all three V1 loop glycans. A PGT135, B PG9, C PGT145, D PGT151, E HIVIG (HIV hyperimmune globulin) and F PGV04. Figure S6. Residues in V3 are important for promiscuous binding of the mannose-patch. V3 residues in A IAVI C22 N332A, B IAVI C22 N334 and C 6545.v4.c1 were mutated to confer neutralization by the PGTs125-131. Numbers represent IC50 values for each antibody and are reported as µg/ml. [file 12977_2016_241_MOESM1_ESM.pptx]

## Slide 1
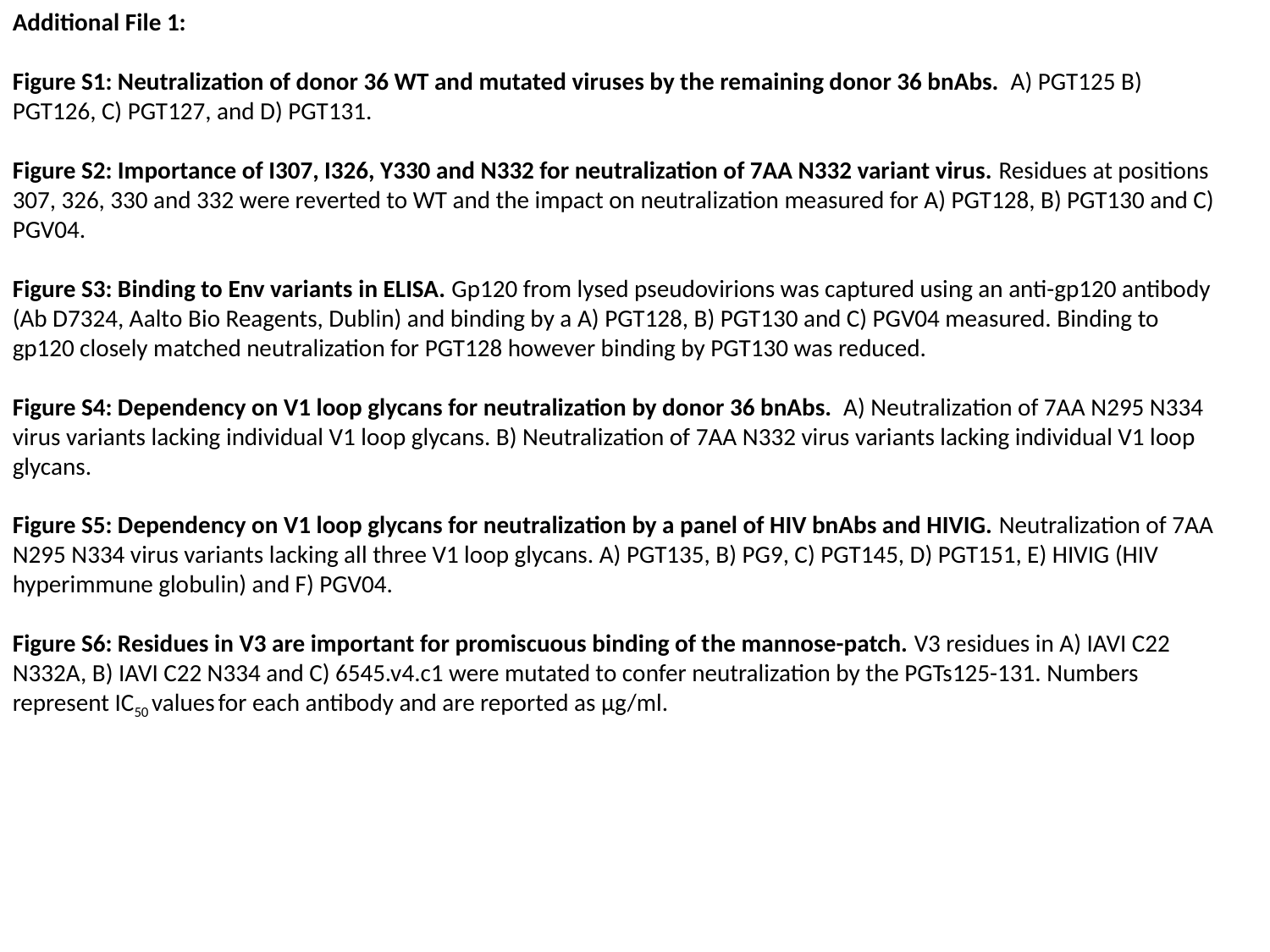

Additional File 1:
Figure S1: Neutralization of donor 36 WT and mutated viruses by the remaining donor 36 bnAbs. A) PGT125 B) PGT126, C) PGT127, and D) PGT131.
Figure S2: Importance of I307, I326, Y330 and N332 for neutralization of 7AA N332 variant virus. Residues at positions 307, 326, 330 and 332 were reverted to WT and the impact on neutralization measured for A) PGT128, B) PGT130 and C) PGV04.
Figure S3: Binding to Env variants in ELISA. Gp120 from lysed pseudovirions was captured using an anti-gp120 antibody (Ab D7324, Aalto Bio Reagents, Dublin) and binding by a A) PGT128, B) PGT130 and C) PGV04 measured. Binding to gp120 closely matched neutralization for PGT128 however binding by PGT130 was reduced.
Figure S4: Dependency on V1 loop glycans for neutralization by donor 36 bnAbs. A) Neutralization of 7AA N295 N334 virus variants lacking individual V1 loop glycans. B) Neutralization of 7AA N332 virus variants lacking individual V1 loop glycans.
Figure S5: Dependency on V1 loop glycans for neutralization by a panel of HIV bnAbs and HIVIG. Neutralization of 7AA N295 N334 virus variants lacking all three V1 loop glycans. A) PGT135, B) PG9, C) PGT145, D) PGT151, E) HIVIG (HIV hyperimmune globulin) and F) PGV04.
Figure S6: Residues in V3 are important for promiscuous binding of the mannose-patch. V3 residues in A) IAVI C22 N332A, B) IAVI C22 N334 and C) 6545.v4.c1 were mutated to confer neutralization by the PGTs125-131. Numbers represent IC50 values for each antibody and are reported as µg/ml.

## Slide 2
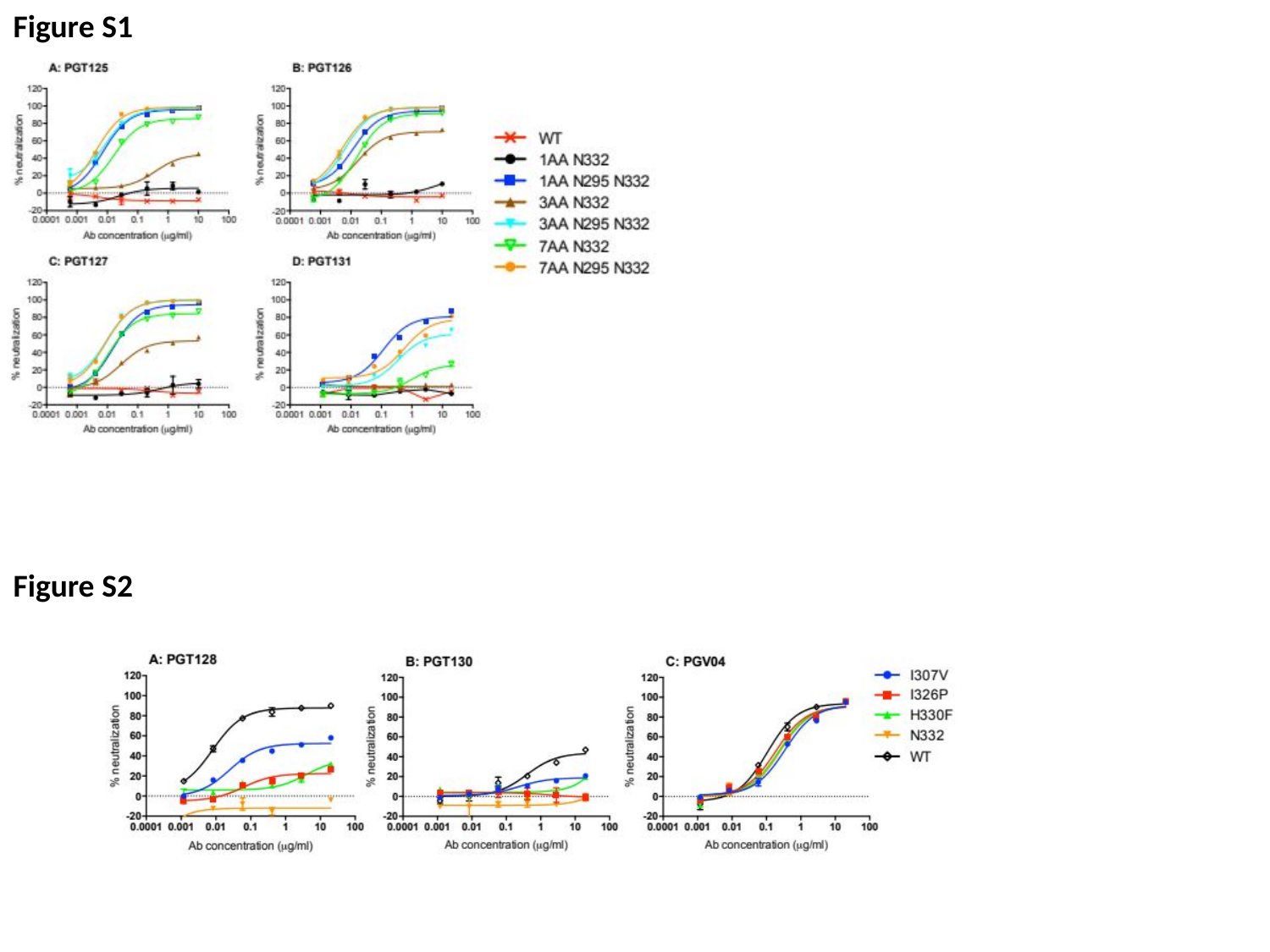

Figure S1
Figure S2

## Slide 3
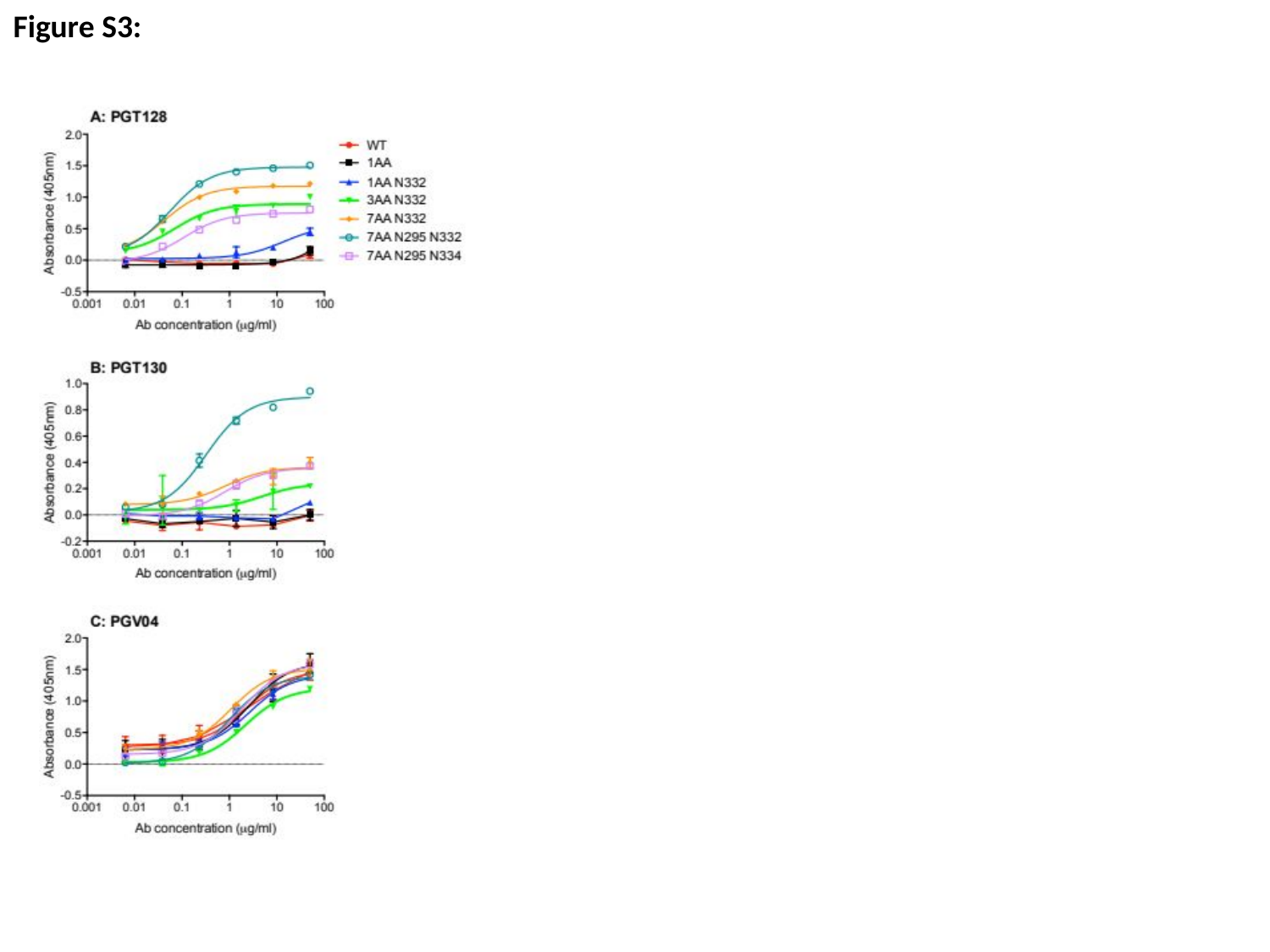

Figure S3:

## Slide 4
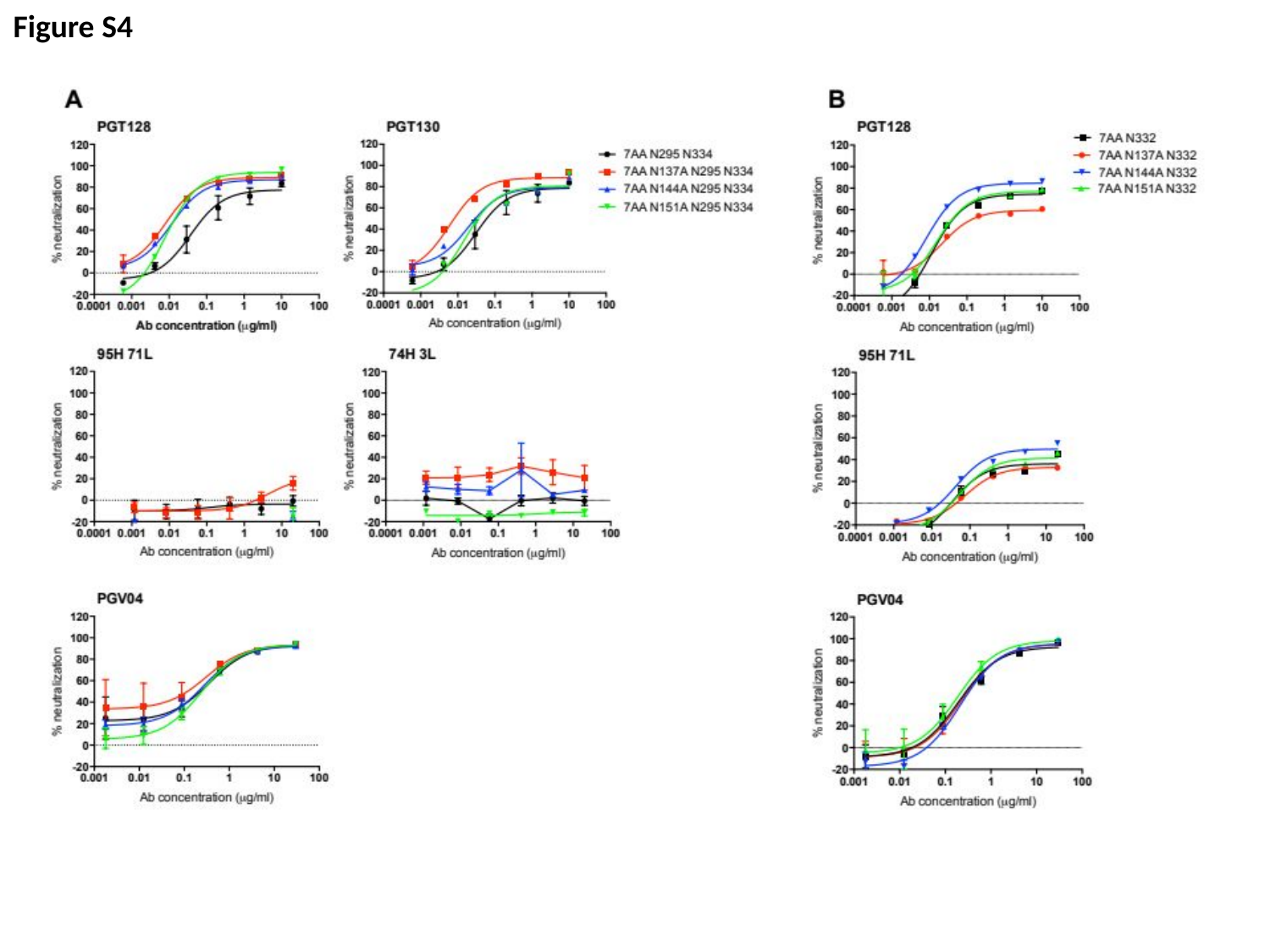

Figure S4

## Slide 5
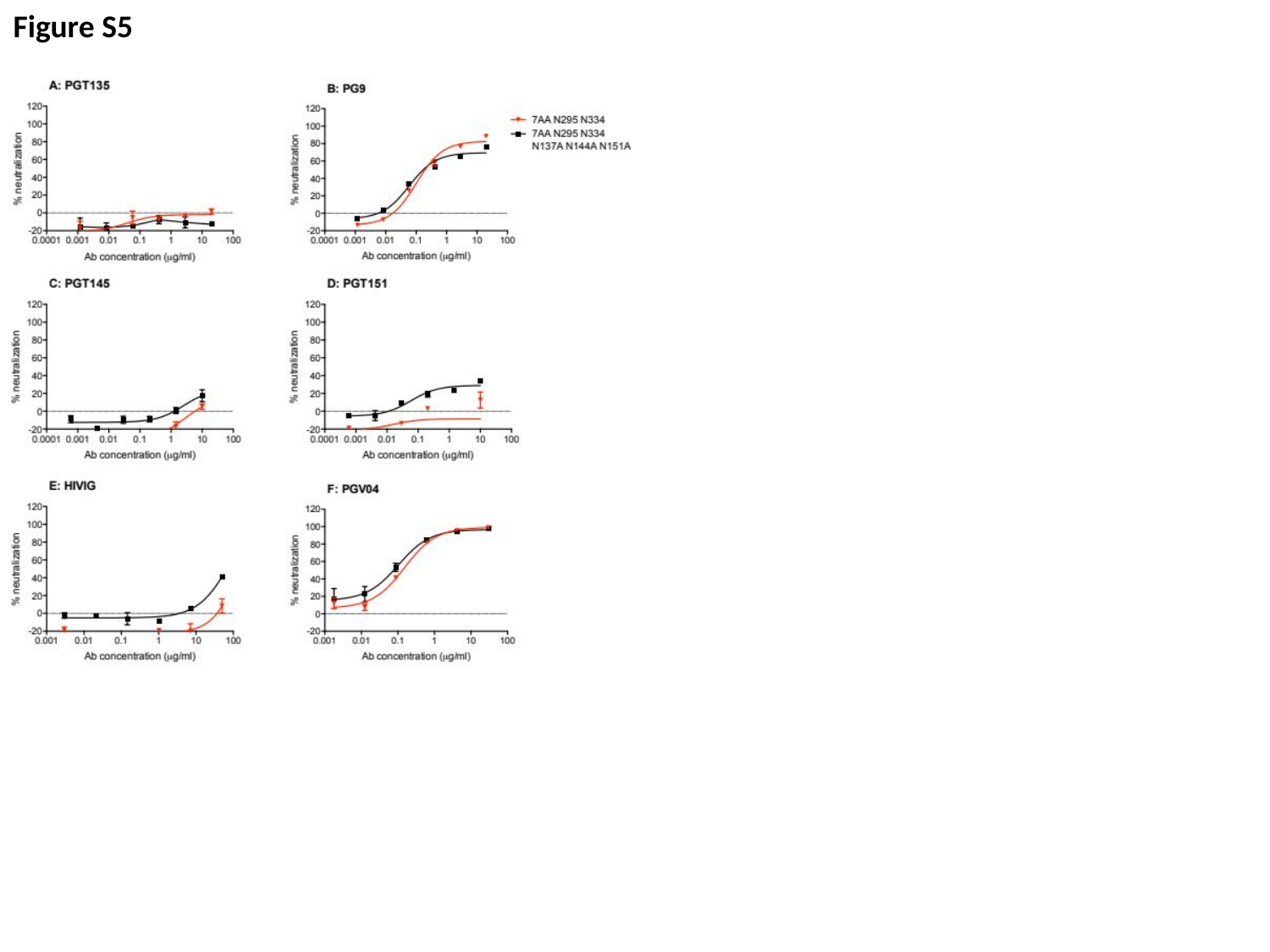

Figure S5

## Slide 6
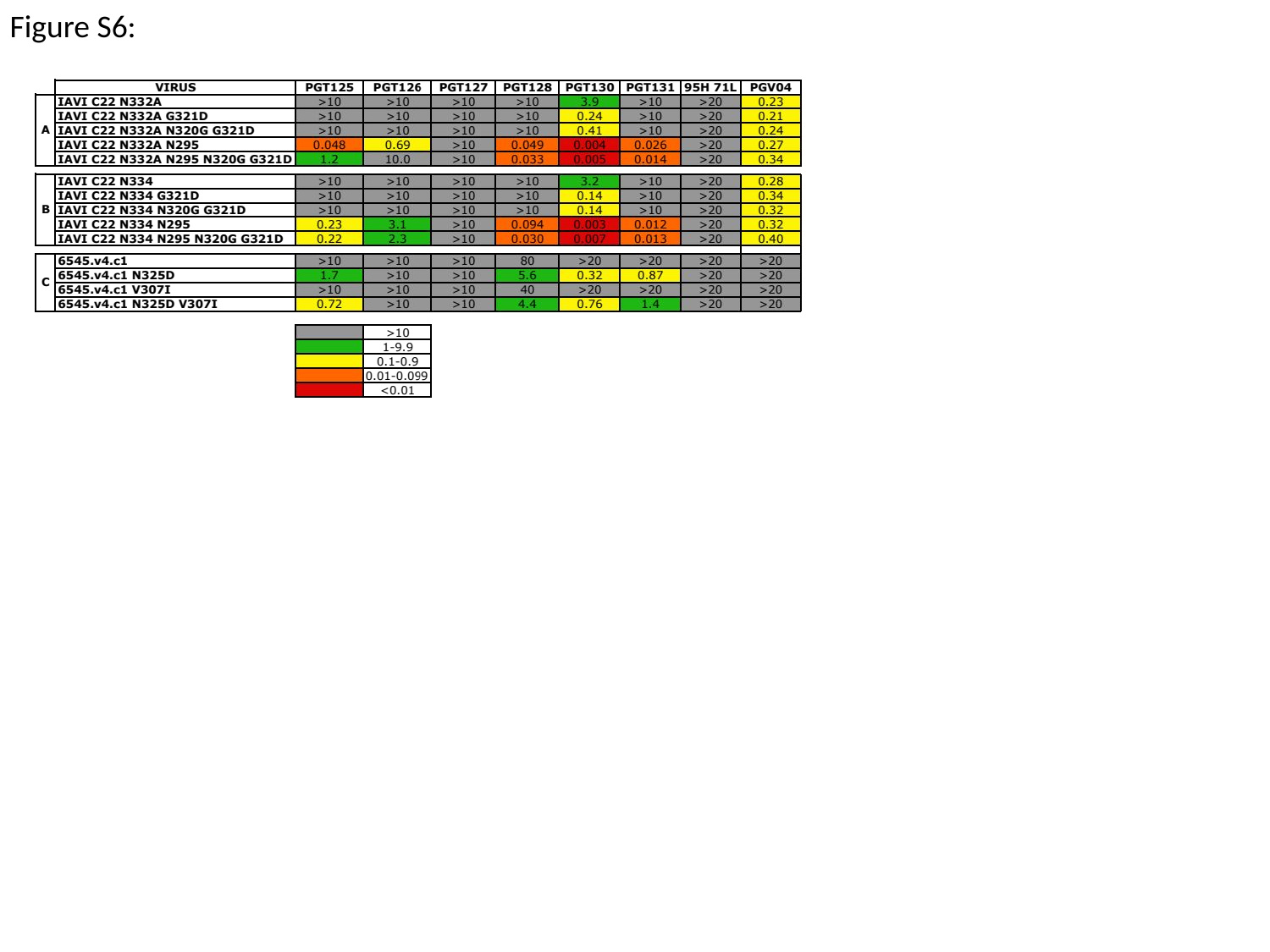

Figure S6:
